# Nosocomial bloodstream infection and venovenous extracorporeal membrane oxygenation: a retrospective cohort study

**DOI:** 10.1186/cc12063

**Published:** 2013-03-19

**Authors:** B Serra de Oliveira, S Mendes Fernandes, C França

**Affiliations:** 1 Hospital de Santa Maria CHLN, Lisboa, Portugal

## Introduction

Venovenous extracorporeal membrane oxygenation (VV-ECMO) for respiratory failure in the ICU is used in a variety of clinical situations and has been demonstrated to significantly improve survival without disability in adult respiratory distress syndrome [1]. ECMO has been presented as a risk factor for bloodstream infection although recently published data do not support this view or the use of antibiotic prophylaxis [2]. We aimed to examine VV-ECMO as a risk factor for nosocomial bloodstream infection.

## Methods

A retrospective cohort study from patients admitted to our ICU between April 2009 and June 2012. We compared incidence rates of nosocomial bacteremia using the Hospitals in Europe Link for Infection Control through Surveillance (HELICS) between general ICU and VV-ECMO patients and used multiple logistic regression analysis to control for possible confounders.

## Results

During the study period 1,146 patients were admitted and 16 received VV-ECMO. The incidence of bloodstream infection in patients with ECMO was 19/1,000 exposure-days versus 4,9/1,000 exposure-days in general ICU patients (incidence rate ratio of 3.82; 95% CI: 2.0 to 7.3; *P <*0.001). Bacteremia was mostly due to Gram-negative agents (65%). The patients with bloodstream infections under ECMO (*n *= 10) had a nonsignificant younger age (*P *= 0.08) and a lower SAPS II score (*P *= 0.03) compared with non-ECMO patients (*n *= 25). VV-ECMO patients had a significantly higher risk of primary bacteremia than non-ECMO patients (*P *= 0.04). Patients with bloodstream infections in the VV-ECMO group had a lower crude mortality rate (OR: 0.1, *P *= 0.04), not confirmed in the adjusted analysis. There were no crude or adjusted differences in the time to bacteremia or infections due to multiple drug-resistant microorganisms (OR: 0.26; *P *= 0.085) between groups.

## Conclusion

This study suggests that VV-ECMO patients have a significantly higher risk for primary nosocomial bloodstream infection. A larger study is needed to confirm such findings and to assess the need for specific intervention, namely routine antibiotic prophylaxis.
